# Meta-Chirality: Fundamentals, Construction and Applications

**DOI:** 10.3390/nano7050116

**Published:** 2017-05-17

**Authors:** Xiaoliang Ma, Mingbo Pu, Xiong Li, Yinghui Guo, Ping Gao, Xiangang Luo

**Affiliations:** State Key Laboratory of Optical Technologies on Nano-Fabrication and Micro-Engineering, Institute of Optics and Electronics, Chinese Academy of Sciences, P. O. Box 350, Chengdu 610209, China; maxl@ioe.ac.cn (X.M.); pmb@ioe.ac.cn (M.P.); qiling006@163.com (X.L.); guoyinghui8@163.com (Y.G.); gaop@ioe.ac.cn (G.P.)

**Keywords:** chiral metamaterials, circular dichroism, optical activity, extrinsic chirality, reconfigurable

## Abstract

Chiral metamaterials represent a special type of artificial structures that cannot be superposed to their mirror images. Due to the lack of mirror symmetry, cross-coupling between electric and magnetic fields exist in chiral mediums and present unique electromagnetic characters of circular dichroism and optical activity, which provide a new opportunity to tune polarization and realize negative refractive index. Chiral metamaterials have attracted great attentions in recent years and have given rise to a series of applications in polarization manipulation, imaging, chemical and biological detection, and nonlinear optics. Here we review the fundamental theory of chiral media and analyze the construction principles of some typical chiral metamaterials. Then, the progress in extrinsic chiral metamaterials, absorbing chiral metamaterials, and reconfigurable chiral metamaterials are summarized. In the last section, future trends in chiral metamaterials and application in nonlinear optics are introduced.

## 1. Introduction

Though symmetry brings out special beauty in the areas of architecture, arts, and physics, asymmetry is still the main melody in our universe. From the basic components of living things, such as DNA and proteins, to the celestial bodies such as galaxies, asymmetry is everywhere. Chirality just refers to the structures or geometries that have no symmetric plane and cannot superpose to their mirror images. Due to the breaking of symmetry, extraordinary properties can be obtained in electromagnetic chiral structures which are quite different from achiral ones. Typical electromagnetic properties are circular dichroism (CD) and optical activity (OA). CD represents different transmission or absorption between the right-handed circularly polarized (RCP) and the left-handed circularly polarized (LCP) waves, while OA stands for the rotation of a polarization plane when a linearly-polarized light transmits through chiral structures. These special optical properties can be found in natural chiral materials, including sugar solutions and quartz crystals [1].

The optical activity of chiral materials was first discovered by Dominique Arago in 1811 [1]. He found that the polarization of linearly polarized sunlight was rotated off its original state when it passes through a quartz crystal; the outgoing polarization can be characterized by another polarizer. Optical activity has played an important role in analytical chemistry, crystallography, and molecular biology [2]. It has also been used to detect life forms in space missions, as biological molecules are mainly chiral and would lead to distinctly different physiological responses with different spatial configurations [2]. The optical rotation and circular dichroism of natural materials are rather weak. To efficiently make use of these two properties, thick materials are required, which leads to bulk volume devices and systems, and is not compatible with on-chip optical instruments.

Bulky metamaterials and two-dimensional metamaterials (metasurfaces) are artificial materials composed of different materials or structures. By properly designing the structure type and parameters of the units, metamaterials present exotic properties that cannot be found in natural materials; for example, negative refraction index [3,4,5,6,7,8,9] and anti-Doppler effect [10]. The periodical unit cells give rise to giant interaction between the electromagnetic wave and the artificial structures. Besides, the interaction can be artificially controlled by tuning the type and size of the unit cells to achieve extraordinary electromagnetic functions, such as high-efficiency absorption [11,12,13,14,15,16,17], broadband polarization conversion [18,19,20,21,22,23,24,25,26,27], and optical imaging breaking through the diffraction limit [28,29,30,31,32]. Furthermore, the thickness and size of the fabricated devices would be greatly decreased, which provides an opportunity to manipulate the electromagnetic radiation in the sub-wavelength scale, and could be beneficial for highly integrated microwave or optical systems [33]. The related theories in metamaterials or metasurfaces for generalized reflection or refraction, absorption, and polarization manipulation has greatly enlarged the application realm of traditional electromagnetic laws [34,35].

As a typical type of metamaterial, a chiral metamaterial has special unit cells that lack mirror symmetry and present CD and OA in macroscopy. In order to achieve giant chirality, series of chiral unit cells are designed, including twisted cross structures [36,37], Slavic symbols [38,39,40], multi-layered arc structures [41,42,43,44,45], twisted split rings [46,47,48,49,50,51], Y-shape structures [52,53], and helix structures [54,55,56,57,58]. Due to the strong coupling between the electric and magnetic fields in chiral metamaterials, the resulting chirality can be much larger than that in natural chiral materials, thus providing an opportunity to realize negative refraction index, which has been theoretically and experimentally proved by Pendry [59] and Monzon [60], independently of each other. Besides in the microwave range, chiral metamaterials in terahertz [61,62,63], infrared [64,65], and visible range [66,67,68] have also been constructed. These chiral metamaterials present giant potential value in biological detection, chemical analysis, optical force, and optical display.

In this review, we outline the past achievement of chiral metamaterials, together with the fundamental theory, constructing methods, and applications. The review is arranged as follows. In Section 2, we analyze the fundamental theory of chiral materials. The construction methods are reviewed in Section 3. In Section 4, Section 5 and Section 6, several kinds of chiral metamaterials are discussed, including extrinsic chiral metamaterials, absorption chiral metamaterials, and reconfigurable chiral metamaterials, respectively. Finally, a conclusion and perspective of future development for chiral metamaterials is proposed.

## 2. Fundamentals of Chiral Media

As the lack of mirror symmetric, the coupling between the electric and magnetic fields exists in chiral materials or structures, which is different from anisotropic mediums. The classical constitutive relationship equations have variation in the basis form compared to traditional Maxwell equations, as shown in Formula (1), which is proposed by Post [69].

(1) $$\{\begin{array}{c} \vec{D}=\varepsilon \vec{E}+j\xi \vec{H}\\ \vec{H}=\vec{B}/\mu +j\xi \vec{E} \end{array}$$

In the constitutive formula, ε is the permittivity and *μ* is the permeability, respectively. The electric displacement vector $\vec{D}$ is related to both the electric field $\vec{E}$ and magnetic field $\vec{B}$. This is also true for the magnetic induction intensity $\vec{H}$. The parameter ξ represents the coupling strength between the electric and magnetic fields, and is named as the chiral parameter. When it equals to 0, the constitutive equation has the same form as normal isotropic materials.

Combing the above formula with source-free Maxwell equations, the wave equation of an electric field in chiral materials can be derived as follows.

(2) $$\begin{array}{l} \nabla \times \vec{E}=j\omega \vec{B}=\omega \left(\vec{D}-\varepsilon \vec{E}\right)/\xi =j\nabla \times \vec{H}/\xi -\omega \varepsilon \vec{E}/\xi \\ =j\nabla \times \left(\vec{B}/\mu +j\xi \vec{E}\right)/\xi -\omega \varepsilon \vec{E}/\xi =-\nabla \times \vec{E}+\frac{1}{j\omega \mu }\nabla \times \nabla \times \vec{E}-\omega \varepsilon \vec{E}/\xi \end{array}$$

where *ω* stands for the frequency.

Equation (2) can be transformed as follows.

(3) $$\nabla \times \nabla \times \vec{E}-2\omega \mu \xi \nabla \times \vec{E}-\omega^{2}\mu \varepsilon \vec{E}=0$$

It is well-known that the vector calculation formula has the below formation for a passive electric field.

(4) $$\nabla \times \nabla \times \vec{E}=\nabla \left(\nabla \cdot \vec{E}\right)-\nabla^{2}\vec{E}=-\nabla^{2}\vec{E}$$

Thus, it can be concluded that the wave equation of $\vec{E}$ and $\vec{H}$ in chiral materials has the following form.

(5) $$\begin{array}{l} \nabla^{2}\vec{E}+2\omega \mu \xi \nabla \times \vec{E}+\omega^{2}\mu \varepsilon \vec{E}=0\\ \nabla^{2}\vec{H}+2\omega \mu \xi \nabla \times \vec{H}+\omega^{2}\mu \varepsilon \vec{H}=0 \end{array}$$

The electric field is defined as $\vec{E}=E_{0}\exp(-j\omega t+j\vec{k}\cdot \vec{r})$, where $\vec{k}$ is the wave vector and $\vec{r}$ is the position vector. Then, Equation (3) can be transformed into Equation (6).

(6) $$-\vec{k}\times \vec{k}\times \vec{E}-j2\omega \mu \xi \vec{k}\times \vec{E}-\omega^{2}\mu \varepsilon \vec{E}=0$$

Subsequently, it can be concluded that the wave number *k* satisfies the following equation:

(7) $$k^{2}={\left(\frac{\omega^{2}\mu \varepsilon -k^{2}}{2\omega \mu \xi }\right)}^{2}$$

The above equation has two eigensolutions in the forms of circular polarization, and the wave numbers can be written as follows.

(8) $$\{\begin{array}{c} k_{R}=\omega \mu \xi +\omega \sqrt{\mu \varepsilon +\mu^{2}\xi^{2}}\\ k_{L}=-\omega \mu \xi +\omega \sqrt{\mu \varepsilon +\mu^{2}\xi^{2}} \end{array}$$

The subscripts “R” and “L”, respectively, stand for the RCP and LCP waves.

The above analysis demonstrates that the RCP and LCP waves have different propagating constant in the chiral medium. Accordingly, the refraction index of the chiral material can be approximately represented as $n_{\pm }=\sqrt{\varepsilon \mu }\pm \xi$, where the subscript “+” stands for RCP and “–“ for LCP. It is notable that when the chiral parameter is large enough, the refraction index can be negative even *ε* and *μ* are positive, and this is the main reason for the great attention paid to chiral materials in recent years. As the refraction indices are unequal, the transmit amplitude and phase have discrepancy between these two circular polarizations, resulting in circular dichroism and optical activity.

In general, the optical rotation can be represented with polarization rotation angle *γ* with the formula $\gamma =\left(n_{+}-n_{-}\right)\pi d/\lambda_{0}$, where *λ*_0_ is the wavelength in vacuum, and *d* is the thickness of the chiral material. To simplify the simulation and calculation, the rotation angle can also be calculated by the transmit phase of the two circular polarizations. That is, $\gamma =\left[\arg\left(T_{+}\right)-\arg\left(T_{-}\right)\right]/2$, where *T*_+_ and *T*_−_ are, respectively, the complex transmission for RCP and LCP waves.

When the image parts of the refraction indices of circular polarizations are different, the absorption or transmittances have discrepancy after passing through the chiral material, namely the circular dichroism. The strength of CD can be represented by the ellipticity expressed as

$$\eta =\frac{1}{2}\sin^{-1}\left(\left({\left|T_{+}\right|}^{2}-{\left|T_{-}\right|}^{2}\right)/\left({\left|T_{+}\right|}^{2}+{\left|T_{-}\right|}^{2}\right)\right)$$

It is clear that the polarization rotation angle and ellipticity of the chiral material are directly related to the transmission *T*_+_ and *T*_−_ which can be measured experimentally and simulated using commercial electromagnetic simulation software. The circular transmissions can also be calculated using Jones matrices [70]. However, the effective refraction indices of circular polarizations cannot be directly measured or calculated using the transmission efficiencies, but need a parameter retrieving method with the participation of transmission and reflection in both circular polarizations [71,72,73]. Similar to the parameter retrieval process of anisotropic metamaterials, the effective parameters of refraction index and chirality of chiral metamaterials can be calculated using the parameter retrieval method.

## 3. Construction of Chiral Metamaterials

From the definition of chirality, we know that it lacks mirror symmetry in chiral structures. Therefore, the destination of the construction of chiral metamaterial becomes simple, through the breakage of the mirror symmetry of ordinary three-dimensional structures.

**Figure 1:**
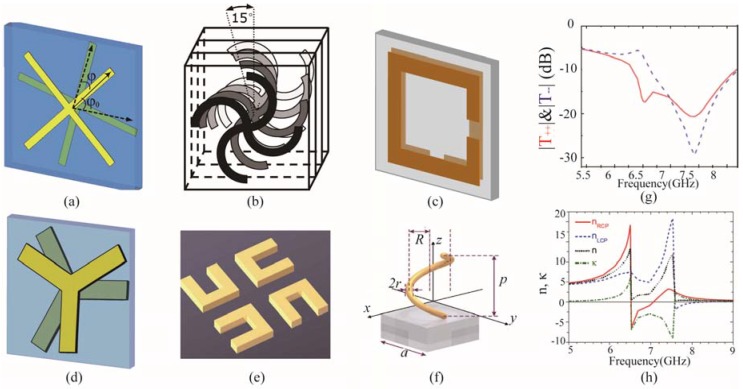
Typical unit cells of chiral metamaterials. (**a**) Twisted cross structure [74]; (**b**) Twisted gammadion structure; (**c**) Twisted split ring structure; (**d**) Twisted Y-shape structure; (**e**) Twisted U-shape structure; (**f**) Helix structure; (**g**)The circular transmission for the chiral metamaterial in (**a**); (**h**) The retrieved effective refractive index and chirality for the chiral metamaterial in (**a**). (**a**,**g**,**h**) are reproduced with permission from [74]. Copyright American Physical Society, 2009; (**b**) is reproduced with permission from [75]. Copyright American Physical Society, 2009; (**e**) is reproduced with permission from [77]; and (**f**) is reproduced with permission from [55].

Figure 1 depicts a series of typical units of chiral metamaterials with different structures. Figure 1a demonstrates a two-layer chiral metamaterial unit cell, which is composed of two isotropic twisted cross structures with a twisted angle *φ* around its normal axis [74]. When the angle does not equal to integer multiples of π/2, the structure has no mirror plane and presents chiral character. As shown in Figure 1b, multiple layers of gammadion structure with a twist angle between neighboring layers make up a chiral unit, which presents circular dichroism in two distinct resonances [75]. This construction method of twisting layered symmetric structures with a certain angle has produced fruitful applications, including twisted split rings [76] and Y-shaped [52] chiral metamaterials shown in Figure 1c,d, which respectively present broadband optical activity and multiband chirality. This constructing method can be further developed. Figure 1e is the unit cell of the chiral metamaterial, whose basic unit is composed of four twisted U-shape metallic structures with a neighboring twist angle of π/2 [77]. Since the U-shape structure is anisotropic, the 90 degree twisted composite presents chirality and can be used to manipulate the polarization states. Furthermore, this kind of U-shape chiral metamaterial usually has multi-layers, and the U-shape structures in the same location in neighboring layers also have a π/2 twist angle.

The above-mentioned chiral metamaterials are constructed by twisting multiple layered anisotropic or isotropic structures to achieve symmetry breaking. Differently, a spiral helix has intrinsic chirality for its continuous stereo-structure, which has been employed to achieve broadband polarization conversion in microwave [78,79] and terahertz range [80,81].

The chiral unit cells depicted in Figure 1a–e would superpose with themselves when the unit cells are rotated by π/2 about their normal axis. These kinds of chiral metamaterials are called four-fold symmetric structures, or C4 symmetric structures. In these kinds of chiral metamaterials, no conversion exists between the circular polarizations when passing through the chiral metamaterials. With a linearly polarized incidence, a maximum circular transmission of –3 dB would be obtained. Besides, negative refractive index has been realized using these chiral metamaterials shown in Figure 1a,b. As seen in the scheme of the unit cell, the rotation handedness of the chiral structures directly determines the chirality, and thus leads to the negative refractive index for left- or right-handed circular polarization. The giant circular dichroism of Figure 1a results in the discrepancy of circular transmission, as shown in Figure 1g [74]. Using the standard parameter retrieval process, the effective refractive index and chirality were obtained, as depicted in Figure 1h, which is associated directly with the transmission spectra.

**Figure 2:**
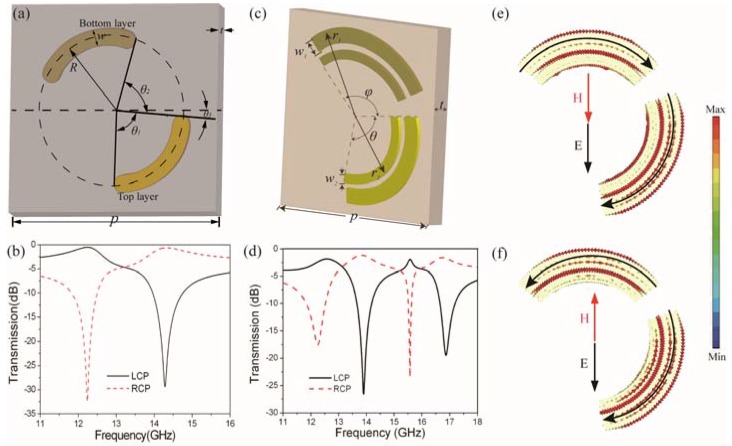
Chiral metamaterials based on twisted arc structures. (**a**) Unit cell with two twisted arcs; (**b**) Circular transmission spectra of (**a**) with a linearly-polarized incidence; (**c**) Unit cell with two pairs of twisted arcs; (**d**) Circular transmission spectra of (**c**) with linearly-polarized incidence; (**e**,**f**) The surface current distribution of the chiral metamaterial in (**c**) at the first and second resonances, respectively. (**a**,**b**) are reproduced with permission [42]. Copyright IEEE, 2014; (**c**–**f**) are reproduced with permission [43]. Copyright AIP Publishing LLC, 2012.

Actually, some chiral metamaterials without C4 symmetry have been proposed as a polarizer with higher efficiency, as shown in Figure 2 [42,43]. Due to the lack of C4 symmetry and mirror symmetry, these chiral metamaterials have circular dichroism and polarization conversion at the same time. By properly designing the structural parameters, the polarization conversion may contribute positively to the transmission of certain circular polarization, leading to the near unit polarization conversion ratio with a linearly polarized incident wave. This may greatly increase the polarization conversion efficiency.

Figure 2a shows the unit cell of a chiral metamaterial which converts the incident linearly polarized wave into different circularly-polarized waves with loss less than 0.6 dB, as shown in Figure 2b [42]. The unit cell is composed of two twisted arcs printed on two sides of a dielectric substrate. The arc on the bottom layer is twisted by an angle of θ_2_ with respect to the arc on the top layer. When a linearly polarized wave is normally incident on the chiral metamaterial, it can be decomposed into two circularly polarized waves with equal amplitude and phase. As the unit cell lacks C4 symmetry, the two circular polarizations partially convert to their cross-polarizations at the resonance. Furthermore, the converted cross polarizations have opposite functions to the transmission of the original circular polarizations. For example, the converted LCP wave from the incident RCP wave is in phase with the transmitted LCP wave, which results in constructive interference and enhances the LCP transmission at the lower resonance. However, the converted RCP is out of phase with the transmitted RCP wave, and this would lead to deconstructive interference with the transmitted RCP wave, leading to the transmission valley of the RCP wave. Furthermore, the operating resonance can be increased by properly adding more arcs into the unit cell [41,43]. Figure 2c,d demonstrate a multi-band circular polarizer based on two pairs of twisted arcs with different sizes, which converts the incident linearly polarized wave into different circularly-polarized waves with low loss and high extinction ratio at four distinct resonances [43]. The distribution of the surface current reveals the coupling between the electric and magnetic fields well. As shown in Figure 2e,f, the surface current distributions at the lower two resonances of the multi-band chiral metamaterial in Figure 2c shows opposite electromagnetic coupling, which leads to opposite chirality and opposite circularly-polarized radiation, agreeing well with the transmission spectra in Figure 2d.

This mechanism of combining chirality with anisotropy creatively provides an efficient method of increasing the polarization conversion efficiency in chiral metamaterials. These twisted arc chiral metamaterials have inspired the construction of terahertz polarization converts [44] and nonlinear optical devices [45].

## 4. Extrinsic Chirality in Planar Metamaterials

The main character of chiral metamaterials lies in their non-symmetrical structures. However, recent studies show that some planar metamaterials with symmetric unit cells can also present chirality in oblique illumination states, known as extrinsic chirality [82,83,84,85]. Essentially, this extrinsic chirality comes from the spatial asymmetry of the metamaterials.

**Figure 3:**
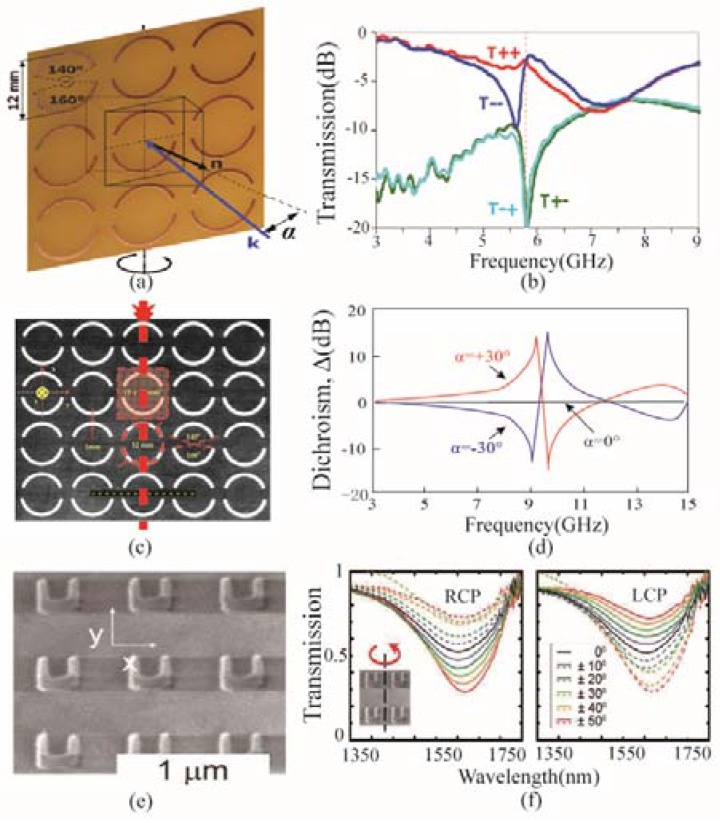
Extrinsic planar chiral metamaterials and their transmission spectra under oblique incidence. (**a**) Extrinsic planar chiral metamaterial with split rings; (**b**) Circular transmission and circular polarization conversion spectra of the split ring metamaterial with 30° rotation angle; (**c**) Planar metamaterial with split ring apertures; (**d**) Circular dichroism spectra of the metamaterial in (**c**) with ±30° rotation angles; (**e**) Planar metamaterial with U-shape unit cells; (**f**) Transmission spectra of right-handed circular polarization (RCP) and left-handed circular polarization (LCP) with different rotation angle of the unit in (**e**). (**a**,**b**) are reproduced with permission [82]. Copyright AIP Publishing LLC, 2008; (**c**,**d**) are reproduced with permission [84]. Copyright American Physical Society, 2009; (**e**,**f**) are are reproduced with permission [85]. Copyright American Physical Society, 2012.

Figure 3 depicts the unit cells of extrinsic chiral metamaterials. The metamaterial shown in Figure 3a, is composed of planar metallic spiral arcs on a substrate board. The central angles of the two arcs in each unit are 140° and 160° [82]. The arcs have a symmetric axis along the vertical direction. When the metamaterial is normally illuminated by circularly polarized electromagnetic waves, it presents anisotropy without chirality. Nevertheless, keeping the direction of the incident wave unchanged, the metamaterial would present chirality while rotating the metamaterial around the symmetric axis shown in Figure 3a. Figure 3b shows the transmission (*T*_++_ and *T*_−−_)) and polarization conversion (*T*_+−_ and *T*_−+_) spectra of the circularly polarized waves with the rotation angle of 30°. The first subscript stands for the radiated polarization, and the second subscript for the incident polarization. The symbol “+” represents the RCP while “_−_” represents the LCP. It is clear that the metamaterial demonstrates circular dichroism at two distinct frequencies, where obvious discrepancy between the circular transmission spectra is observed.

In Figure 3c, the complementary structure of unit cell in Figure 3a is presented [84]. The chiral metamaterial is composed of period of split ring apertures in a metallic board, and is also anisotropic with electromagnetic wave normally impinging onto it. By rotating the metamaterial around the diagonal axis (the red dashed line depicted in Figure 3c, the chiral character would be reversed. When the rotation angel equals to ±30°, the circular dichroism spectra are opposite to each other, as shown in Figure 3d, which demonstrates obvious chirality of the metamaterial in the oblique situation. Figure 3e is another example of extrinsic chiral metamaterial with single layer of U-shape unit [85]. With the oblique incident angle changing in the range from −50° to 50°, the opposite varying trend of the RCP and LCP transmission can be observed, and CD gets larger when the incident angle is increased. It can be seen that the transmission of these two circular polarizations coincide at 0° incident angle, which validates the intrinsic achiral character of the metamaterial.

The above examples show that even in symmetrical structures, chirality can still be achieved by rotating the structures around a certain axis. Because this chirality does not originate from the intrinsic unit cell structure, it is called extrinsic chirality.

Aside from the extrinsic chirality of the unit cells of the metamaterial, some metasurfaces have also presented analogical properties by arranging their anisotropic unit cells in a chiral way [86,87,88,89,90,91,92]. These chiral distributed metasurfaces usually have 2D structures and have the advantages of ultrathin thickness and ultra-light weight compared to traditional optical lenses. The basic unit cells have different structures and spatial distributions to achieve various optical functions. In these metasurfaces, geometry phase, rising from the process of polarization conversion in anisotropic subwavelength structures, is utilized to control the wave front of the incident light [93]. The varying trend of the geometry phase is opposite for different circular polarizations. Therefore, different field distributions would be present when the LCP and RCP light are incident upon the metasurface, which is analogous to the circular dichroism of chiral materials and can be categorized as extrinsic chiral metamaterials.

**Figure 4:**
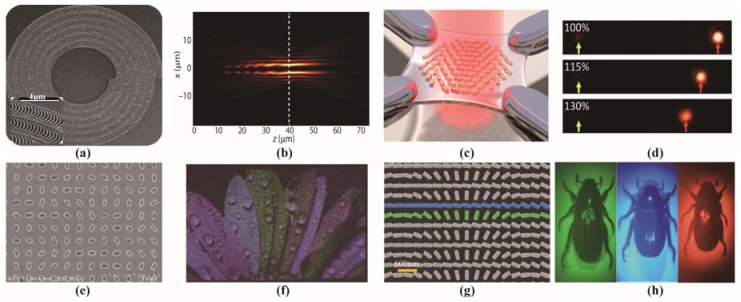
Different functional metasurfaces with chiral distribution of anisotropic unit cells. (**a**) SEM photo of metasurface with spirally distributed catenary structure for Bessel beam generation; (**b**) Electric field of the generated Bessel beam; (**c**) Stretchable metasurface with active focus length based on the chiral distribution of metallic nano rods; (**d**) The focus position of the metasurface with different stretch ratios; (**e**) Metasurface to generate a hologram with chirally-distributed slits; (**f**) The whole color hologram photo produced by the metasurface in (**e**); (**g**) Planar lens based on metamaterial with chirally arranged dielectric posts; (**h**) Multiple images of beetle *Chrysina gloriosa* using the metasurface in (**g**). (**a**,**b**) are reproduced with permission [94]; (**c**,**d**) are reproduced with permission [95]. Copyright American Chemical Society, 2016; (**e**,**f**) are reproduced with permission [97].

Figure 4a demonstrates the spirally distributed catenary structures to generate a high-order Bessel beam, and the produced light beam is shown in Figure 4b [94]. The catenary structures can be found in biological tissues like nepenthes. It is a typical example of natural structures inspiring optical devices. With an RCP planar light illumination, the transmitted light beam is converted to a high-order Bessel beam. Nevertheless, the transmitted light would emanate with an LCP incidence. Besides, the continuous phase manipulation of this catenary structure has been employed to achieve the perfect generation of light beams carrying orbital angular momentum. Figure 4c,d depict a stretchable extrinsic chiral metamaterial, and its focus lengths can be tuned with different strain ratios of the substrate by force [95]. The metasurface is composed of rectangular nanorods with chiral distribution. Similarly, the focusing function of this metasurface is still sensitive to the rotation direction of the incident circularly polarized light. Figure 4e,f shows a chiral metamaterial for producing a full-color hologram and the corresponding generated holograms. The metasurface shown in Figure 4e is constructed by periodic elliptical holes with chiral distribution on a single layer of metallic membrane [96]. With certain circularly-polarized incidence, the hologram of leaves with RGB color was obtained, as seen in Figure 4f. It is also proved that an opposite hologram can be realized when the incident light has cross polarization. Figure 4g is the SEM image of a dielectric chiral metasurface used as an imaging lens at multiple wavelengths. The unit cells of the metasurface are rectangular TiO_2_ dielectric posts arranged with chiral phase distribution [97]. As shown in Figure 4h, using this planar lens, images of the beetle *Chrysina gloriosa* can be observed at distinct wavelength covering the visible range. This kind of metasurface with chiral unit cell distribution has great potential value in on-chip optical systems.

## 5. Chiral Metamaterials for Absorption

Theoretical analysis of chiral media reveals that the refraction indices of LCP and RCP have discrepancy at the resonant frequencies, which is the basis of optical activity and circular dichroism. According to the above-mentioned fundamental theory of chiral media, we know that circular dichroism originates from the difference between the image part of circular polarization refraction indices, and leads to different absorption between the circular polarizations while passing through the chiral medium, which indicates that chiral media can be utilized as absorbers for certain circular polarization and promise new applications in detecting, imaging, stealth, and communication systems.

**Figure 5:**
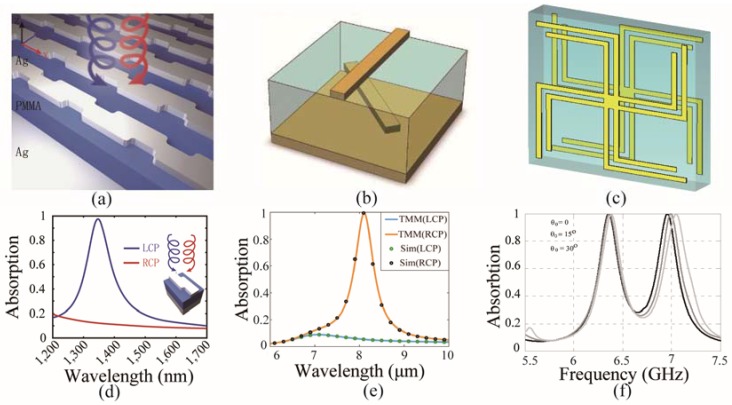
Chiral metamaterials for absorption. (**a**) Schematic structure of planar chiral metamaterial for selective circular polarization absorption; (**b**) Unit cell of chiral metamaterial with two layers of twisted metallic rods; (**c**) Scheme of chiral unit with dual band absorption; (**d**) Circular polarization absorption spectra of the chiral metamaterial in (**a**); (**e**) Numerical results of circular polarization absorption spectra (solid lines calculated by transfer matrix method (TMM) and dot lines calculated by CST software) of the chiral metamaterial in (**b**); (**f**) Absorption spectra of the chiral metamaterial in (**c**). (**a**,**d**) are reproduced with permission [98]. Copyright Nature Publishing Group, 2015; (**b**,**e**) are reproduced with permission [99]. Copyright American Chemical Society, 2016; (**c**,**f**) are reproduced with permission [100]. Copyright Progress in Electromagnetics Research Symposium, 2013.

Li et al. proposed a chiral absorber and achieved giant circular dichroism in the near-infrared range [98]. The schematic structure of the chiral metamaterial is shown in Figure 5a, where periodic metallic Z-shape chiral units are deposited on Ag substrate with a polydimethylsiloxane (PMMA) spacer. Obvious discrepancy in circular absorptions were obtained when the circularly-polarized light normally impinged onto it, as depicted in Figure 5d. It was observed that the incident LCP light was nearly perfectly absorbed, while the absorption of the RCP was less than 10%. Upon combining this chiral absorber with an active material (e.g., a semiconductor), tunable absorption character can be obtained with bias DC voltage stimuli. Figure 5b shows another chiral metamaterial with two layers of twisted metallic rod in each unit [99]. At the wavelength of 8 μm, the incident RCP light is perfectly absorbed while the LCP light is reflected, demonstrating giant circular dichroism, as seen in Figure 5e. Furthermore, this chiral absorption can be realized in a wide angle range up to ±80°. The above two chiral metamaterials with selective absorption for circularly polarized light may have potential applications in biology detection, display, and sensor devices.

Besides the selective absorption character, chiral metamaterials are suitable to construct multi-band absorbers due to their multiple modes of cross-coupling between the electric and magnetic fields. As shown in Figure 5c, the twisted structure with two branches in each arm is a variant of gammadion [100]. The introduction of the extra branches can increase the resonant frequency bands of the metamaterial, and has been widely used in the design of metamaterials. When this chiral metamaterial is applied as an absorber, it achieves multi-band near-perfect absorption of the incident linearly-polarized waves. Figure 5f depicts the absorption spectra of this chiral metamaterial, in which two separate absorption peaks can be observed. The absorption remains well when the incident angle varies by ±30°. It is concluded that the chiral metamaterial provides an efficient method to construct absorbers with various destinations.

## 6. Reconfigurable Chiral Metamaterials

**Figure 6:**
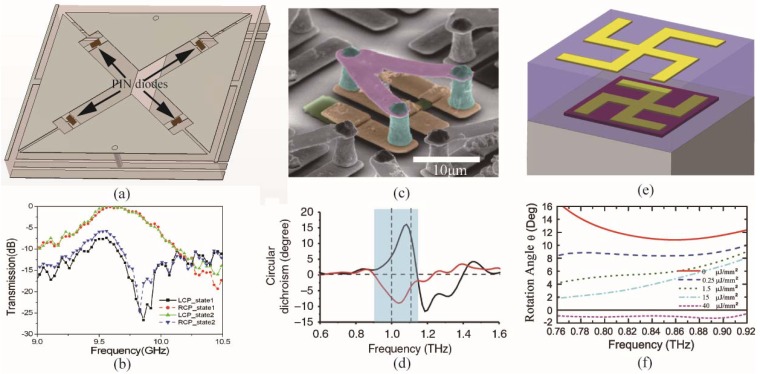
Reconfigurable chiral metamaterial. (**a**) Scheme of the unit cell of chiral metamaterial with four PIN diodes loaded; (**b**) The circular transmission spectra of the chiral metamaterial in (**a**) with different working states of the diodes; (**c**) Three-dimensional reconfigurable chiral metamaterial with silicon pad (green part); (**d**) Circular dichroism of the chiral metamaterial in (**c**) when it is illuminated by light or not; (**e**) Reconfigurable gammadion shape chiral metamaterial with semiconductor substrate (red part); (**f**) Rotation angle of the chiral metamaterial in (**e**) under different illumination intensities. (**a**,**b**) are reproduced with permission [105]. Copyright John Wiley and Sons, 2014; (**c**,**d**) are reproduced with permission [109]. Copyright Nature Publishing Group, 2012; (**e**,**f**) are reproduced with permission [110]. Copyright American Physical Society, 2012.

It has been desired to actively tune the electromagnetic properties of chiral metamaterials, which is also the key function to achieve intelligent electromagnetic devices. Nevertheless, it remains a large challenge to construct active chiral metamaterials, due to the need to invert the handedness of the asymmetric structures. In recent years, active materials such as semiconductors [101,102], phase change materials [103], and micro-electro-mechanical systems (MEMS) [104] have been introduced into the design process of chiral metamaterials to obtain reconfigurable chirality and controllable electromagnetic properties. In the microwave range, active devices such as positive-intrinsic-negative (PIN) diodes and varactors have been employed to construct active chiral metamaterials [105]. By controlling the outer bias voltage, the working states of the diodes can be switched on and off. In the structural scheme, the diodes can be analogous to a metallic strip or a dielectric gap in the unit cell. Therefore, it has the ability to rebuild the structure of the metamaterial. Figure 6a depicts the unit cell of the reconfigurable chiral metamaterial in microwave range [105]. It is composed of metallic structures with rectangular and cross apertures in the bottom and top layers, respectively. In the cross aperture, four PIN diodes are loaded and divided into two groups with opposite working states. Therefore, the handedness of the chiral metamaterial can be switched by exchanging the working states of the PIN diodes, leading to the opposite circular transmission character, as shown in Figure 6b.

Active devices have been widely used in microwave active metamaterials for their tunable character [106,107]. However, they are not suitable for higher frequency metamaterials due to their millimeter-scale volume. In metamaterials with operating frequency higher than terahertz, phase-change materials, semiconductor materials, and MEMS are common active materials to construct reconfigurable chiral metamaterials. Yin et al. employed a layer of phase-change material Ge_3_Sb_2_Te_6_ between two stacked nano-rods to form a reconfigurable chiral metamaterial [108]. The refraction index of Ge_3_Sb_2_Te_6_ in the mid-infrared range would change from 3.5 + 0.01*i* to 6.5 + 0.06*i* when it was heated up to 160 °C. With thermal control, a large spectral shift of circular dichroism reaching 18% was realized. Kenanakis et al. proposed several chiral structures based on Si which can be transformed from insulating to conducting state through photo-excitation, to achieve giant tunable optical activity in terahertz band [102]. Zhang et al. introduced a 3D chiral metamaterial with Si pads (green parts) in the unit cell, as shown in Figure 6c [109]. When the metamaterial was illuminated by near-infrared laser pulses with an intensity of about 1 mJ/cm^2^, the conductivity of Si could be sharply increased to a highly conducting state, leading to the chirality switching of the metamaterial. The reversion of the circular dichroism of the chiral metamaterial can be obtained in Figure 6d, proving that the chirality character is switched from left-handed to right-handed. Figure 6e depicts a planar chiral metamaterial with gammadion shape [110]. In this chiral metamaterial, semiconductor Si (purple square in Figure 6e was employed as the substrate of the chiral metamaterial, and dynamic control of the chirality was accomplished by exciting photocarriers in the intrinsic silicon islands using near-infrared femtosecond laser pulses. The rotation angle of the chiral metamaterial could be tuned from 12° to near 1° as the laser power increased from 0 to 40 μJ/mm^2^, as demonstrated in Figure 6f.

Due to the intrinsic chirality in biological molecules (e.g., DNA), some 3D chiral structures were fabricated using the DNA origami method [111,112,113]. In this method, metallic nanoparticles are precisely decorated to the DNA strands by assembly process [114]. The resulted structures have giant chirality due to the plasmon effect of the metallic nanoparticles. Furthermore, these artificial chiralities based on DNA origami method can also be switched, either controlling the handedness of the twisted direction of nanoparticles or aligning the direction of the fabricated structure to be perpendicular or parallel to the light beam. This switchable chirality category has obvious advantage in biological sensing.

## 7. Conclusions and Outlook

Chiral metamaterials have developed rapidly in recent years, and the operating frequencies have risen from the microwave to the optical range. The progress in fundamental theory and fabrication technology has accelerated the development of chiral metamaterials and resulted in fruitful applications in polarization manipulating, imaging, and detecting areas. The above chiral metamaterials have been reviewed, as have the two main characters, namely circular dichroism and optical activity. Meanwhile, chiral metamaterials also have other attractive properties, such as optical nonlinearity [115,116], asymmetric transmission [117], and optical force [118,119]. In recent years, the concept of chirality has been enlarged from media and structures to electromagnetic fields, and asymmetric electromagnetic fields such as circularly polarized light are also known as chiral and are named as superchiral light [120].

It can be expected that chiral metamaterials will get more applications in biological detection because most biological molecules and tissues are chiral. Crossing and integration of disciplines would help chiral metamaterials go further. However, the study of chiral metamaterials still needs detailed investigation—both in theory and fabrication technology—to enhance the strength of chirality. Broadband and high efficiency would be the main focus for the future direction of chiral metamaterials. Furthermore, the design of reconfigurable chiral metamaterials is still a challenge, and should be paid enough attention for the increasing requirement in active polarization manipulation in smart detection and communication systems.
